# The Efficacy and Safety of the Combination Therapy With GLP-1 Receptor Agonists and SGLT-2 Inhibitors in Type 2 Diabetes Mellitus: A Systematic Review and Meta-analysis

**DOI:** 10.3389/fphar.2022.838277

**Published:** 2022-02-04

**Authors:** Chen Li, Jie Luo, Mingyan Jiang, Keke Wang

**Affiliations:** ^1^ Department of Pharmacy, The First Hospital of China Medical University, Shenyang, China; ^2^ School of Pharmacy, China Medical University, Shenyang, China

**Keywords:** glucagon-like peptide-1 receptor agonists, sodium-glucose co-transporter-2 inhibitors, combination therapy, type 2 diabetes mellitus, meta-analysis

## Abstract

**Aims:** Glucagon-like peptide-1 receptor agonists and sodium-glucose co-transporter-2 inhibitors play a key role in the treatment of type 2 diabetes mellitus. This meta-analysis aims to evaluate the efficacy and safety of their combination, emphatically focusing on the effects of treatment duration and add-on drugs.

**Methods:** Seven databases were searched until June 2021 for randomized controlled trials with a duration of at least 12 weeks, evaluating the effects of combination therapy with glucagon-like peptide-1 receptor agonists and sodium-glucose co-transporter-2 inhibitors.

**Results:** A total of eight eligible articles were included, pooling data retrieved from 1895 patients with type 2 diabetes mellitus. Compared to monotherapy, combination therapy resulted in a greater reduction in glycated haemoglobin (HbA1c), body weight, fasting plasma glucose (FPG), 2 h postprandial glucose (2 h PG), systolic blood pressure (SBP), body mass index (BMI) and low-density lipoprotein cholesterol (LDL-C). The decrease in HbA1c, body weight and FPG was maintained for more than 1 year, but these effects gradually regressed over time. The risk for hypoglycaemia was significantly increased with combination therapy. In addition, drug discontinuation, diarrhoea, injection-site-related events, nausea, vomiting and genital infections were more likely to occur in combination therapy.

**Conclusion:** Glucagon-like peptide-1 receptor agonist and sodium-glucose co-transporter-2 inhibitor combination therapy showed superior effects on reducing HbA1c, body weight, FPG, 2 h PG, SBP, BMI and LDL-C, without major safety issues, when compared with monotherapy in patients with type 2 diabetes mellitus.

## 1 Introduction

Type 2 diabetes mellitus, also called non-insulin-dependent diabetes mellitus, is a chronic metabolic disease characterized by high blood glucose levels, caused by beta cell dysfunction and insulin resistance, and the majority of patients are adults. WHO data show that the incidence of type 2 diabetes has increased dramatically over the past few decades (WHO, 2016). Long-term disease may cause macrovascular and microvascular complications that seriously affect the quality of life of patients (Zheng et al., 2018).

In recent years, an increasing number of drugs have been widely used in the clinic, including glucagon-like peptide-1 receptor agonists (GLP-1RAs) and sodium-glucose co-transporter-2 inhibitors (SGLT-2is). GLP-1RAs are a class of drugs that can activate the GLP-1 receptor and promote its binding to the specific receptor of pancreatic β-cells, ultimately improving insulin concentration, inhibiting glucagon secretion, reducing food intake, and delaying gastric emptying (Drucker et al., 2017). SGLT2is are a novel class of oral glucose-lowering drugs that can inhibit renal resorption of glucose and increase the excretion of urine glucose (Brown et al., 2019). Some clinical studies have demonstrated that SGLT2is can also help patients reduce weight, improve blood lipids, protect kidneys and reduce the risk of cardiovascular events (Monami et al., 2014; Zinman et al., 2015). In the guidelines issued by the American Association of Clinical Endocrinologists (AACE), American Diabetes Association (ADA) and Chinese Diabetes Society, GLP-1RAs and SGLT-2is are the choice drugs in the case of metformin failure (Garber et al., 2019; Jia et al., 2019; Buse et al., 2020).

During the progression of T2DM, it is quite difficult to control blood glucose because the patients also present with metabolic diseases, such as obesity, dyslipidaemia and hypertension. It is therefore conceivable that patients with a long history of T2DM and more complications will use a variety of antihyperglycaemic drugs, especially novel SGLT-2i and GLP-1RA drugs (Jia et al., 2019). Their different glucose-lowering mechanisms may also produce complementary synergistic effects (Busch and Kane, 2017). Therefore, GLP-1RA and SGLT-2i combination therapy may have better clinical efficacy and safety. However, the potential additional benefits of this combination therapy are still uncertain (Li et al., 2021), and the meta-analysis or systematic reviews currently reported are not comprehensive enough. In this meta-analysis, we aimed to summarize all the relevant randomized controlled trails (RCTs) that had results and evaluate the safety and efficacy of GLP-1RA and SGLT-2i combination therapy compared to monotherapy.

## 2 Materials and Methods

This systematic review and meta-analysis has been performed in accordance with the Preferred Reporting Items for Systematic Reviews and Meta-Analyses (PRISMA) statement (Hutton et al., 2015).

### 2.1 Search Strategy

Seven electronic databases such as PubMed, Web of Science, Cochrane Library, Embase, CNKI, SinoMed and Wanfang Data were searched from their inception to June 2021 with no language restrictions. Unpublished clinical trials were also identified by searching ClinicalTrials.gov. The search terms are “glucagon-like peptide-1 receptor agonist” (including exenatide, liraglutide, albiglutide, lixisenatide, semaglutide, dulaglutide, taspoglutide), “sodium glucose cotransporter 2 inhibitor” (including canagliflozin, empagliflozin, dapagliflozin, ipragliflozin, luseogliflozin, tofogliflozin, remogliflozin, sergliflozin, sotagliflozin, ertugliflozin) and “type 2 diabetes mellitus”. These terms were adjusted to fit the relevant rules in each database.

### 2.2 Study Selection

Trials were deemed eligible for inclusion if they 1) were RCT design; 2) compared the efficacy and safety of GLP-1RA and SGLT-2i combination therapy to monotherapy; 3) had a follow-up of at least 12 weeks; 4) included only adult subjects (age ≥18) with T2DM; 5) included complete key clinical data, such as glycated haemoglobin (HbA1c), fasting plasma glucose (FPG), adverse events (AEs) and, etc.; 6) no restrictions on gender, race or nationality. Non-RCT designed trials, duplicate reports, case reports, trials without results and trials included ineligible patients, such as children, adolescents and patients without diabetes or with type 1 diabetes, were excluded.

### 2.3 Data Extraction

Data were extracted by two authors (Chen Li and Jie Luo) independently using a pilot tested form containing the following information: 1) publication information (first author, year of publication); 2) study information (study name, study type, clinicaltrials.gov trial number (NCT ID), follow-up period, sample size, and inclusion criteria of target population); 3) the baseline information of participates [age, sex ratio, diabetes duration, body mass index (BMI), weight, systolic blood pressure (SBP), diastolic blood pressure (DBP), HbA1c, and FPG]; 4) intervention information (classes of study drugs, doses); 5) end-points, including efficacy outcomes [HbA1c, body weight, FPG, 2 h postprandial glucose (2 h PG), SBP, DBP, waist circumference, and lipid levels] and safety outcomes (hypoglycaemia, nausea, diarrhoea, vomiting, injection site-related events, urinary tract infection, genital infection etc.).

### 2.4 Quality Assessment

The risk of bias was assessed by two independent authors (Chen Li and Jie Luo) using the Cochrane risk-of-bias tool (Sterne et al., 2019). The assessment included following seven aspects: random sequence generation, allocation concealment, blinding of participants and personnel, blinding of outcome assessment, incomplete outcome data, selective reporting and other bias (funding). Each item was assigned as low, unclear or high risk of bias. Discrepancies were resolved through discussion or by a third reviewer (Keke Wang).

### 2.5 Data Synthesis and Analysis

The primary outcome of this meta-analysis was the change in HbA1c from baseline to the final follow-up. The secondary outcomes included the changes in body weight, FPG and SBP from baseline to the final follow-up and the incidence of adverse events, such as hypoglycaemia, nausea and genital infection. The differences in continuous outcome variables, such as HbA1c and body weight, were calculated by standardized mean difference (SMD) with 95% confidence intervals (CIs). Dichotomous variables, such as hypoglycaemia or other adverse events, were analysed by relative risks with 95% CIs. If the standard deviation (SD) was unreported, it was calculated according to the standard error (SE) or the 95% CI. For some studies with three arms, we divided them into two observations based on different experimental groups and compared them to the same control group.

Heterogeneity between these results were accessed using I^2^ statistic. If the I^2^ was over 50%, the heterogeneity was considered as high and the inverse variance heterogeneity random effects model would be used to analyse. Otherwise, the fixed effects model was used. Besides, subgroup analysis and sensitivity analysis were carried out to explore the sources of heterogeneity. Publication bias has been evaluated by Egger’s test. All statistical analyses were carried out using Stata 14.0 software (StataCorp LP), and were performed at the 0.05 significance level.

## 3 Results

### 3.1 Study Selection and Characteristics

**FIGURE 1:**
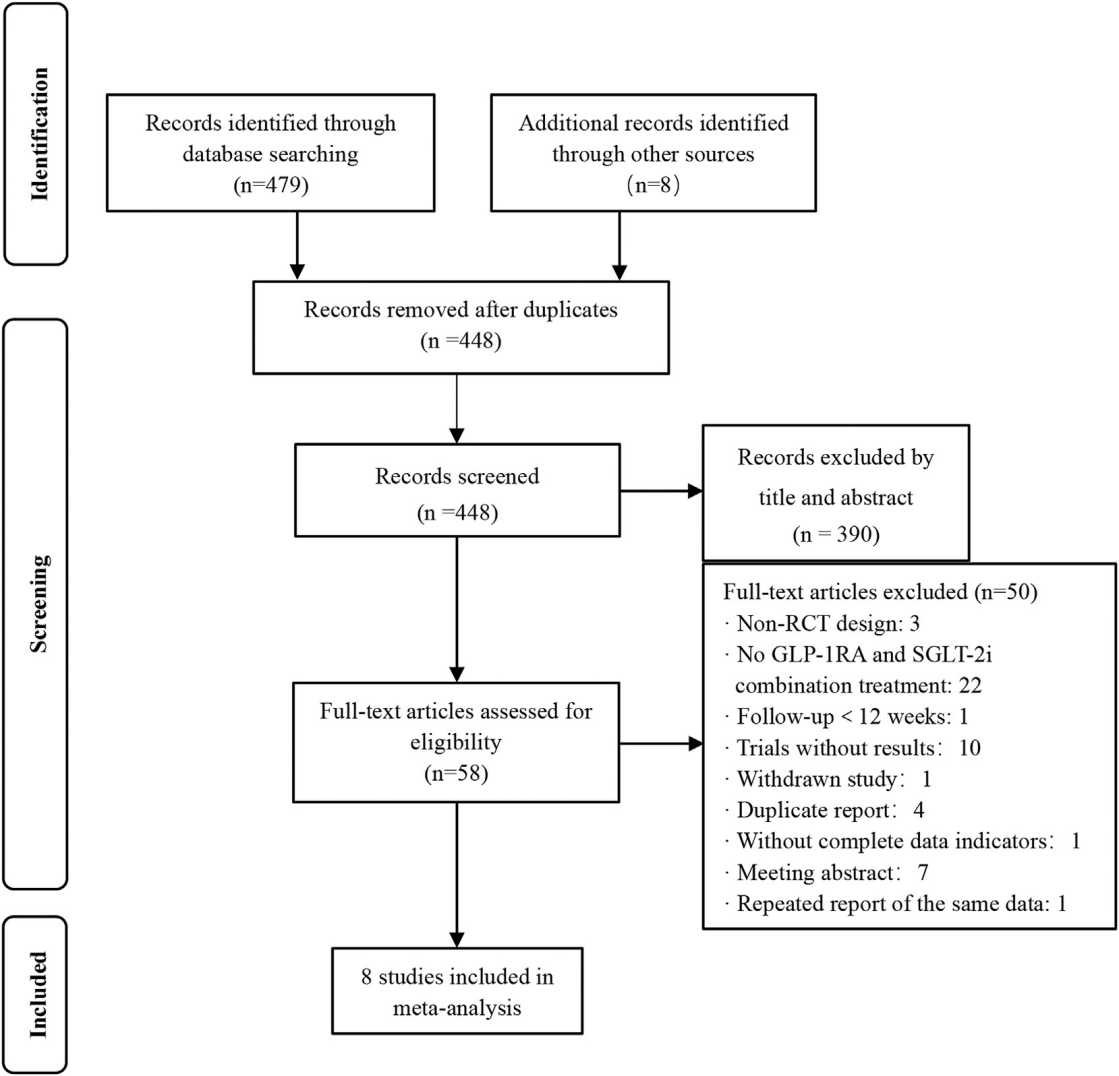
Flow chart of the study selection. Non-RCT design: non-randomized controlled trial design.

In total, 479 articles were identified in the primary search from databases, and eight additional studies were identified through other sources (published meta-analysis and review). Among them, in addition to 39 duplicates, there were 390 unrelated studies, while 50 studies were discarded for the following reasons: non-RCT designs, no GLP-1RA and SGLT-2i combination treatment, follow-up <12 weeks, trials without results, withdrawn studies, duplicate reports, without complete data indicators, meeting abstracts and repeated reports of the same data. Only 8 articles met the inclusion criteria. The flowchart of study selection is presented in Figure 1.

There were 1895 patients included in our final 8 studies, and their baseline characteristics are shown in Table 1. These studies were all randomized controlled trials that were reported from 2018 to 2021. The baseline characteristics of these patients were assessed, and there were no significant differences between arms (ES 0.00; 95% CI: -0.04, 0.03; *p* = 0.924). The sample size of these studies ranged from 30 to 695, and the proportion of males ranged from 47.9 to 66.7%. The mean age ranged from 52.3 to 60.1 years, the mean diabetes duration ranged from 6.6 to 9.9 years, the mean HbA1c level ranged from 7.5 to 9.3%, the mean FPG ranged from 8.80 to 10.8 mmol/L, the mean BMI ranged from 31.3 to 34.9 kg/m^2^, the mean SBP ranged from 127.8 to 134 mmHg, and the mean DBP ranged from 77.33 to 82 mmHg. The follow-up durations ranged from 12 to 104 weeks. Seven articles compared GLP-1RA and SGLT-2i combination therapy with SGLT-2i monotherapy, and four articles compared GLP-1RA and SGLT-2i combination therapy with GLP-1RA monotherapy.

**TABLE 1:** Characteristics and baseline of the studies included in the meta-analysis. Cana: canagliflozin; lira: liraglutide; exe: exenatide; dapa: dapagliflozin; dula: dulaglutide; sema: semaglutide; NR: not reported; ①: HbA1c; ②: weight; ③: FPG; ④: SBP; ⑤: DBP; ⑥: 2 h PG; ⑦: BMI; ⑧: waist circumference; ⑨: TG; ⑩: TC; ⑪: HDL-C; ⑫: LDL-C; ⑬: adverse event. *Data are presented as n (%) or mean (SD).

Study	NCT ID	Interventions	Control	Follow-up weeks	Age years	Participants No.	Male n (%)	Diabetes duration years	BMI kg/m^2^	Weight kg	SBP mmHg	DBP mmHg	HbA1c %	FPG mmol/L	Outcome indicators
Ali et al. (2020)	NCT02324842	cana100 mg + lira1.2 mg	cana100 mg; lira1.2 mg	16	52.3 (2.5)	45	24 (53.3)	7.6 (1.5)	34.9 (1.3)	NR	133.7 (3.5)	79.7 (2.7)	8.2 (0.3)	9.8 (0.7)	①②③④⑬
Blonde et al. (2020)	NCT02964247	lira1.8 mg + SGLT-2i	Placebo + SGLT-2i	26	55.2 (10.0)	303	183 (60)	9.9 (7.0)	32.2 (6.1)	91.1 (21.1)	127.8 (13.3)	79.3 (8.9)	8.0 (0.7)	8.9 (2.4)	①②③④⑦⑬
Harreiter et al. (2021)	EudraCT 2016-000574-38	exe2mg + dapa10 mg	Placebo + dapa10 mg	24	60.1 (7.9)	30	20 (66.7)	6.6 (5.0)	31.3 (4.1)	96.5 (17.9)	134 (16)	82 (9)	7.5 (1.0)	8.9 (3.1)	①②③⑦⑧⑨⑪⑫⑬
Jabbour et al. (2018); Jabbour et al. (2020)	NCT02229396	exe2mgQW + dapa10 mg	exe2mgQW + placebo; dapa10 mg + placebo	28, 52, 104	54.3 (9.5)	695	328 (47.9)	7.4 (5.7)	32.7 (6.3)	90.9 (20.7)	129.7 (12.9)	NR	9.3 (1.1)	10.8 (2.8)	①②③④⑥⑬
Ludvik et al. (2018)	NCT02597049	dula1.5 mg + SGLT-2i; dula0.75 mg + SGLT-2i	Placebo + SGLT-2i	24	57.27 (9.36)	424	212 (50)	9.38 (6.16)	32.68 (5.60)	91.49 (20.05)	130.2 (14.62)	77.33 (9.48)	8.04 (0.64)	8.80 (1.85)	①②③⑬
Rongfeng et al. (2020)	NR	dapa10 mg + lira1.8 mg	lira1.8 mg	12	NR	96	51 (53.1)	NR	NR	NR	NR	NR	NR	NR	①③④⑤⑥⑦⑧⑨⑩⑪⑫⑬
Zinman et al. (2019)	NCT03086330	sema1mg + SGLT-2i	Placebo + SGLT-2i	30	57.0 (9.5)	302	176 (58.3)	9.7 (6.1)	31.9 (6.6)	91.7 (21.0)	127.9 (14.5)	78.8 (8.8)	8.0 (0.8)	9.0 (2.1)	①②③④⑤⑦⑧⑨⑩⑪⑫⑬

### 3.2 Quality Assessment

The risk of bias for the included trials is presented in Supplementary Figure S1. Of these articles, one was an open-label study (Ali et al., 2020) that was considered to have a high risk of bias for the blinding of participants and personnel and a high risk of bias for the blinding of outcome assessment. Another study did not state whether it was a double-blind designed trial (Rongfeng et al., 2020), so it was considered to have an unclear risk of bias for the blinding of participants and personnel and an unclear risk of bias for the blinding of outcome assessment. Five articles were funded by pharmaceutical sponsors, who were also involved in the data analysis, so they were considered to have an unclear risk of bias for other bias.

### 3.3 Summary of Outcomes

#### 3.3.1 Efficacy Outcomes

##### 3.3.1.1 HbA1c

Overall, all 8 articles (Jabbour et al., 2018; Ludvik et al., 2018; Zinman et al., 2019; Ali et al., 2020; Blonde et al., 2020; Jabbour et al., 2020; Rongfeng et al., 2020; Harreiter et al., 2021), involving 1895 patients, reported the change in HbA1c from baseline to the final follow-up. Compared to monotherapy, combination therapy showed a more significant reduction in HbA1c by 0.77% (95% CI: -1.03, -0.50; *p* < 0.001) (Supplementary Figure S2), in which the greatest reduction of 1.75% was achieved when semaglutide was added to SGLT-2i monotherapy for 30 weeks.

**FIGURE 2:**
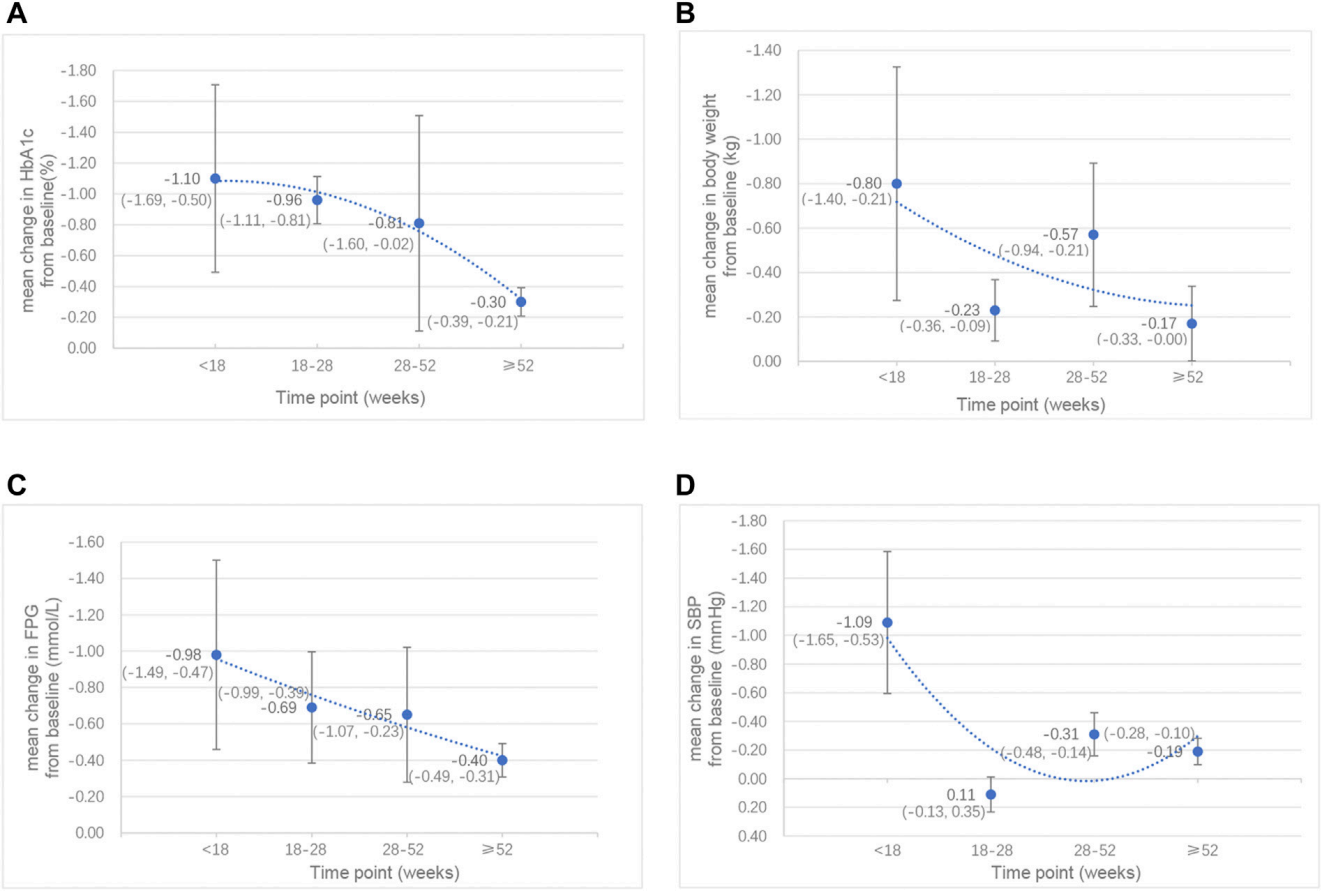
Line chart of the mean changes in **(A)** HbA1c (%), **(B)** body weight (kg), **(C)** FPG (mmol/L) and **(D)** SBP (mmHg) between the combination therapy of GLP-1RA and SGLT-2i and their monotherapy by week. *Data are presented as mean (95% CI).

In addition, we analysed different subgroups such as study duration (Supplementary Figure S3) and add-on therapies, to evaluate the effects of combination therapy on HbA1c levels. According to the study duration of follow-up, we found that the level of HbA1c was significantly decreased and maintained for over 1 year. However, this effect gradually weakened over time. The best control of HbA1c levels was reached within 18 weeks (mean change: −1.10 ± 0.61%), and when the follow-up was from 18 to 52 weeks, the decrease in HbA1c was slightly lower but remained stable. Ultimately, this effect became less significant beyond 1 year (Figure 2A). We also conducted another subgroup analysis on different classes of drugs added to the control group. The results showed that, compared to GLP-1RA monotherapy, the level of HbA1c was reduced by 0.46% (95% CI: −0.77, −0.14; *p* = 0.004) with combination therapy. However, compared to SGLT-2i monotherapy, the level of HbA1c was reduced by 0.91% (95% CI: −1.24, −0.57; *p* < 0.001) with combination therapy, and the reduction was more significant.

##### 3.3.1.2 Other Effect Outcomes

In addition to the significant decrease in HbA1c levels by the GLP-1RA/SGLT-2i combination regimen, we also demonstrated that the FPG levels and the 2 h PG levels of the combination regimen were significantly reduced by 0.66 mmol/L (95% CI: −0.84, −0.47; *p* < 0.001) and 0.33 mmol/L (95% CI: −0.47, −0.20; *p* < 0.001), respectively. This combination regimen was also associated with a greater decrease in body weight (SMD −0.36 kg; 95% CI: −0.50, −0.21; *p* < 0.001) and BMI (SMD −0.96 kg/m^2^; 95% CI: −1.69, −0.23; *p* = 0.010) than their monotherapy. Concerning blood pressure, the results showed that SBP was significantly decreased (SMD −0.33 mmHg; 95% CI: −0.49, −0.17; *p* < 0.001) in the combination therapy group, while there was no significant difference in DBP between combination therapy and monotherapy. However, these effects also decreased with the extension of follow-up time (Figures 2B,C,D). The best effects were reached within 18 weeks, after which these effects were slightly decreased and remained stable during 18–52 weeks and then subsequently became less significant beyond 1 year. Specifically, the effect of body weight was partially recovered by 28–52 weeks, and became less significant again after 1 year; the effect of SBP was recovered by 18–28 weeks, and became less significant again after 28 weeks, although this effect was very slight. Among these outcomes with significant reduction, we found that the greatest reduction in FPG, 2 h PG, BMI and SBP was shown when dapagliflozin was added to liraglutide. In addition, the levels of low-density lipoprotein cholesterol (LDL-C) were significantly reduced with combination therapy (SMD -23.41 mmol/L; 95% CI: −33.74, −13.08; *p* < 0.001). There were no significant effects demonstrated on waist circumference or other blood lipid levels [including the levels of triglycerides (TG), total cholesterol (TC) and high-density-lipoprotein cholesterol (HDL-C)].

The total results for all efficacy measures considered and the results of subgroup analyses according to the class of the drugs are shown in Table 2.

**TABLE 2:** Total results and subgroup results for all efficacy measures that considered of the included studies.

Outcomes	No. of studies (Patients)	Total				Add-in combination		
		SMD	I^2^ (%)	p		SMD	I^2^ (%)	p
HbA1c	8 (1871)	−0.77 (−1.03, −0.50)	94.0	0.000	GLP-1RA	−0.91 (−1.24, −0.57)	94.2	0.000
					SGLT-2i	−0.46 (−0.77, −0.14)	86.2	0.004
Body weight	7 (1778)	−0.36 (−0.50, −0.21)	79.8	0.000	GLP-1RA	−0.34 (−0.54, −0.15)	83.6	0.001
					SGLT-2i	−0.39 (−0.59, −0.19)	63.7	0.000
FPG	8 (1884)	−0.66 (−0.84, −0.47)	87.4	0.000	GLP-1RA	−0.70 (−0.94, −0.46)	88.5	0.000
					SGLT-2i	−0.55 (−0.81, −0.29)	80.2	0.000
SBP	6 (1,431)	−0.33 (−0.49, −0.17)	80.4	0.000	GLP-1RA	−0.21 (−0.39 −0.03)	71.1	0.019
					SGLT-2i	−0.51 (−0.83, −0.19)	86.7	0.002
DBP	2 (398)	−0.86 (−2.19, 0.48)	96.4	0.209	GLP-1RA	−0.19 (−0.42, 0.04)	N/A	0.100
					SGLT-2i	−1.55 (−2.01, −1.10)	N/A	0.000
2 h PG	3 (781)	−0.33 (−0.47, −0.20)	68.7	0.000	GLP-1RA	−0.32 (−0.43, −0.21)	9.9	0.000
					SGLT-2i	−0.37 (−0.63, −0.11)	82.1	0.000
BMI	4 (731)	−0.96 (−1.69, −0.23)	94.2	0.010	GLP-1RA	−0.64 (−1.34, 0.06)	92.8	0.075
					SGLT-2i	−1.95 (−2.44, −1.46)	N/A	0.000
Waist circumference	3 (428)	−1.03 (−2.36, 0.29)	95.8	0.127	GLP-1RA	−0.47 (−0.69, −0.24)	1.4	0.000
					SGLT-2i	−2.46 (−2.99, −1.93)	N/A	0.000
TG	3 (428)	−0.89 (−2.36, 0.58)	96.5	0.236	GLP-1RA	−0.11 (−0.97, 0.74)	80.7	0.792
					SGLT-2i	−2.55 (−3.09, −2.01)	N/A	0.000
TC	2 (398)	−1.27 (−2.59, 0.05)	95.8	0.060	GLP-1RA	−0.61 (−0.84, −0.38)	N/A	0.000
					SGLT-2i	−1.96 (−2.45, −1.47)	N/A	0.000
HDL-C	3 (428)	−2.97 (−6.35, 0.41)	98.7	0.085	GLP-1RA	−10.23 (−30.31, 9.86)	98.2	0.318
					SGLT-2i	2.91 (2.33, 3.49)	N/A	0.000
LDL-C	3 (428)	−23.41 (−33.74, −13.08)	99.7	0.000	GLP-1RA	−35.07 (−103.41, 33.27)	99.8	0.314
					SGLT-2i	−4.19 (−4.91, −3.47)	N/A	0.000

#### 3.3.2 Safety Outcome

We examined the incidence of several safety outcomes, including total adverse events, serious adverse events, adverse events leading to discontinuation, urinary tract infection, deaths, hypoglycaemia, diarrhoea, nausea, injection-site-related events, volume-related events, pancreatic events, acute renal disorders, genital infection, adjudicated cardiovascular events, upper respiratory tract infection, headache, vomiting and ketosis. The total results for all safety outcomes are shown in Table 3.

**TABLE 3:** Total results and subgroup results for all safety measures that considered of the included studies. Exp: experimental.

Outcomes	No. of studies (Patients)	Total			Incidence ratio in Exp. group (%)		Add-in combination		
		RR	I^2^ (%)	p			RR	I^2^ (%)	p
Any AE	5 (1,136)	1.06 (1.00, 1.13)	0.0	0.041	66.44	GLP-1RA	1.08 (1.00, 1.17)	0.0	0.046
						SGLT-2i	1.04 (0.95, 1.13)	0.0	0.399
Any SAE	6 (1765)	0.95 (0.70, 1.29)	0.0	0.743	4.20	GLP-1RA	0.96 (0.65, 1.42)	0.0	0.848
						SGLT-2i	0.93 (0.56, 1.53)	0.0	0.773
AEs leading to discontinuation	5 (1720)	1.44 (1.02, 2.03)	14.8	0.041	4.73	GLP-1RA	1.88 (1.17, 2.99)	9.7	0.008
						SGLT-2i	1.00 (0.59, 1.69)	0.0	0.987
Urinary tract infection	5 (1,070)	1.23 (0.89, 1.70)	0.0	0.214	5.58	GLP-1RA	1.22 (0.78, 1.90)	0.0	0.387
						SGLT-2i	1.24 (0.77, 2.01)	0.0	0.371
Deaths	5 (1,464)	2.64 (0.99, 7.02)	0.0	0.052	0.68	GLP-1RA	2.44 (0.70, 8.43)	0.0	0.159
						SGLT-2i	2.99 (0.61, 14.72)	0.0	0.179
Hypoglycaemia	8 (1890)	1.82 (1.34, 2.47)	0.0	0.000	7.62	GLP-1RA	1.95 (1.32, 2.88)	12.0	0.001
						SGLT-2i	1.62 (0.99, 2.64)	0.0	0.052
Diarrhoea	5 (1720)	1.36 (1.01, 1.83)	37.8	0.040	8.20	GLP-1RA	1.85 (1.27, 2.69)	0.0	0.001
						SGLT-2i	0.75 (0.45, 1.25)	0.0	0.265
Nausea	7 (1861)	1.68 (0.96, 2.93)	77.0	0.069	14.42	GLP-1RA	2.75 (1.61, 4.68)	52.3	0.000
						SGLT-2i	0.67 (0.46, 0.96)	0.0	0.028
Injection-site-related events	4 (769)	1.46 (1.13, 1.87)	0.0	0.003	3.71	GLP-1RA	1.85 (1.26, 2.70)	0.0	0.002
						SGLT-2i	1.19 (0.86, 1.67)	0.0	0.297
Volume-related events	4 (1,418)	0.86 (0.40, 1.85)	0.0	0.695	0.60	GLP-1RA	0.58 (0.23, 1.48)	0.0	0.257
						SGLT-2i	2.49 (0.49, 12.76)	0.0	0.274
Pancreatic events	3 (1,117)	3.44 (0.94, 12.60)	0.0	0.062	0.58	GLP-1RA	5.99 (0.72, 49.76)	0.0	0.097
						SGLT-2i	2.47 (0.48, 12.75)	0.0	0.281
Acute renal disorders	5 (1720)	0.36 (0.16, 0.81)	0.0	0.014	0.58	GLP-1RA	0.41 (0.17, 0.99)	0.0	0.048
						SGLT-2i	0.20 (0.02, 1.70)	0.0	0.140
Genital infection	4 (1,147)	1.18 (0.79, 1.76)	33.5	0.410	3.02	GLP-1RA	0.82 (0.50, 1.33)	0.0	0.424
						SGLT-2i	2.54 (1.19, 5.44)	0.0	0.016
Adjudicated cardiovascular events	4 (1,418)	0.72 (0.40, 1.31)	0.0	0.285	1.66	GLP-1RA	0.72 (0.36, 1.47)	21.5	0.371
						SGLT-2i	0.71 (0.23, 2.22)	0.0	0.558
Upper respiratory tract infection	2 (694)	0.70 (0.49, 1.01)	0.0	0.057	6.49	GLP-1RA	0.66 (0.40, 1.10)	0.0	0.114
						SGLT-2i	0.74 (0.44, 1.26)	0.0	0.270
Headache	5 (1,258)	1.05 (0.77, 1.44)	15.4	0.742	5.37	GLP-1RA	0.88 (0.59, 1.30)	36.6	0.525
						SGLT-2i	1.44 (0.85, 2.43)	0.0	0.173
Vomiting	5 (1816)	1.76 (1.12, 2.77)	49.7	0.014	5.47	GLP-1RA	2.89 (1.58, 5.27)	9.9	0.001
						SGLT-2i	0.66 (0.30, 1.45)	0.0	0.306
Ketosis	3 (593)	0.33 (0.01, 8.10)	N/A	0.499	0	GLP-1RA	N/A	N/A	N/A
						SGLT-2i	0.33 (0.01, 8.10)	N/A	0.499

Overall, the GLP-1RA/SGLT-2i combination regimen was associated with an increased risk for adverse events leading to discontinuation (RR: 1.44; 95% CI: 1.02, 2.03; *p* = 0.041), hypoglycaemia (RR: 1.82; 95% CI: 1.34, 2.47; *p* < 0.001), diarrhoea (RR: 1.36; 95% CI: 1.01, 1.83; *p* = 0.040), injection-site-related events (RR: 1.46; 95% CI: 1.13, 1.87; *p* = 0.003) and vomiting (RR: 1.76; 95% CI: 1.12, 2.77; *p* = 0.014) (Supplementary Figure S4). According to our study, the addition of a GLP-1RA to SGLT-2i treatment demonstrated an increased incidence of adverse events leading to discontinuation (RR: 1.88; 95% CI: 1.17, 2.99; *p* = 0.008), hypoglycaemia (RR: 1.95; 95% CI: 1.32, 2.88; *p* = 0.001), diarrhoea (RR: 1.85; 95% CI: 1.27, 2.69; *p* = 0.001), injection-site-related events (RR: 1.85; 95% CI: 1.26, 2.70; *p* = 0.002), nausea (RR: 2.75; 95% CI: 1.61, 4.68; *p* < 0.001) and vomiting (RR: 2.89; 95% CI: 1.58, 5.27; *p* = 0.001); while the addition of an SGLT-2i to GLP-1RA treatment showed only an increased incidence of genital infection (RR: 2.54; 95% CI: 1.19, 5.44; *p* = 0.016). There was no evidence demonstrating other significant safety issue differences (such as serious adverse events, adjudicated cardiovascular events) between combination therapy and monotherapy.

#### 3.3.3 Sensitivity Analysis

When analysing the changes in the level of HbA1c, we evaluated whether the final analysis was impacted when removing each article in turn. We found that the pooled effect was not significantly changed. Therefore, the results of our analysis were stable.

#### 3.3.4 Publication Bias

Egger’s tests were conducted to detect publication bias. We investigated all the efficacy outcomes that could be calculated, and the findings indicated no evidence of significant publication bias. The results are presented in Supplementary Table S1.

## 4 Discussion

In the present meta-analysis of 8 articles enrolling 1895 patients with T2DM, GLP-1RA/SGLT-2i combination therapy led to a much more significant reduction in glycaemic levels (including HbA1c, FPG and 2 h PG) than monotherapy. In addition, combination therapy was also associated with significant reductions in body weight, BMI and SBP. Nevertheless, with the extension of the follow-up, these therapeutic efficacies weakened. The levels of LDL-C were also significantly decreased in combination therapy. No significant benefit on other efficacy issues was indicated. Compared to monotherapy, the incidence of hypoglycaemia was significantly higher with combination therapy. Combination therapy was also associated with a mildly higher risk for adverse events leading to discontinuation, diarrhoea, injection-site-related events and vomiting. Subgroup analysis on the addition of different drugs to monotherapy demonstrated that the incidence of several adverse events was generally similar, except that the incidences of adverse events leading to discontinuation, hypoglycaemia, diarrhoea, nausea, injection-site-related events and vomiting were mildly increased in the combination treatment when compared with SLGT-2i monotherapy, and the incidence of genital infection was mildly increased when compared with GLP-1RA monotherapy. In addition to the overall analysis on the efficacy and safety outcomes mentioned above, we also focused on the effects of the different classes of drugs as add-ons to monotherapy and the treatment duration on HbA1c, body weight, FPG and SBP, which provided a high level of evidence on the efficacy and safety of GLP-1RA/SGLT-2i combination therapy versus monotherapy in patients with T2DM.

GLP-1RAs and SGLT-2is can effectively control glycaemic levels through different pathways. GLP-1RAs can increase the secretion of insulin and inhibit the secretion of glucagon, as well as increase satiety. SGLT-2is can increase the excretion of urinary glucose by inhibiting SGLT-2 transporters in proximal renal tubules (Tahrani et al., 2016). In addition, SGLT-2is can cause gluconeogenesis, which will counteract the glucose-lowering effect, whereas GLP-1RAs have the opposite effect on gluconeogenesis (Doumas et al., 2018). Therefore, the two drugs have a synergistic effect in controlling blood glucose levels, which is consistent with the results of the present study.

Hypoglycaemia is a common complication of T2DM, and its risk is an important principle for evaluating the safety of treatment options in current diabetes guidelines. Unlike sulfonylureas, GLP-1RAs and SGLT-2is both have a lower risk for hypoglycaemia (Garber et al., 2019). A previous study researched the risk for hypoglycaemia between GLP-1RA/SGLT-2i combination therapy and SGLT-2i monotherapy, and the results demonstrated that it was similar (Castellana et al., 2019). However, according to our analysis, combination therapy had a higher risk for hypoglycaemia, with an RR of 1.82, which was in accordance with the other two studies (Patoulias et al., 2019; Guo et al., 2020). Hence, further research is needed in this area.

Overweight and obesity are quite common in patients with T2DM, and we found that the average body weight of patients who we included was approximately 91.3 kg (excluding the nonreported articles), which is considerably high. SGLT-2is can directly reduce body weight by increasing urinary glucose excretion (Tahrani et al., 2016), whereas the consequent increase in water and energy loss may leading to higher energy usage and an increased appetite (Ferrannini et al., 2015), which in turn leads to increased food intake. Besides, SGLT-2i can also restrict weight loss by regulating inter-organ neural networks that inhibit brown adipose tissue-induced energy expenditure (Chiba et al., 2016). Thus, long-term use of SGLT-2is may reduce the effect of weight loss. Our analysis showed a rapid reduction in weight loss with combination therapy between 18 and 28 weeks, which may be related to the role of SGLT-2is. GLP-1RAs can inhibit appetite, induce thermogenesis of brown adipose tissue and browning adipocyte in white adipose tissue (Beiroa et al., 2014), which might counteract the adverse effect of SGLT-2is on weight loss. Therefore, GLP-1RA and SGLT-2i combination therapy may have a synergistic effect on weight loss, but the effect may decrease over time. This finding was basically consistent with the results of this meta-analysis. Based on the above evidence, short-term GLP-1RA/SGLT-2i combination therapy may be a better option for inadequately controlled, overweight or obese T2DM who are more likely to achieve weight loss goals.

In addition, studies have shown that both GLP-1RAs and SGLT-2is can control blood pressure in T2DM (Sun et al., 2015; Mazidi et al., 2017). Thus, GLP-1RA and SGLT-2i combination therapy may significantly lower blood pressure. The results of our analysis showed a significant reduction in SBP in patients receiving combination therapy, which partly supports the hypothesis mentioned above; however, there was no significant difference in DBP between combination therapy and monotherapy.

Some studies (DeFronzo, 2017; Bertoccini and Baroni, 2021) demonstrated that SGLT-2is can produce osmotic diuresis and natriuresis, improve cardiac load, reduce myocardial oxygen, inhibit myocardial Na^+^/H^+^ exchanger, and finally improve myocardial energetics. And GLP-1RAs can reduce inflammatory markers and regulate endothelial function to play an anti-atherosclerosis and anti-inflammatory role (Garg et al., 2019). However, our results showed that the risk for cardiovascular events was similar between combination therapy and monotherapy. Thus, it remains to be determined whether GLP-1RA/SGLT-2i combination therapy has more benefits on cardiovascular system.

As most GLP-1RAs need to be frequently administered subcutaneously (once weekly, once daily or even twice daily), their long-term usage can cause injection-site-related events (Madsbad, 2016; Nauck et al., 2021), which will lead to lower compliance. According to our analysis, compared to SGLT-2i monotherapy, combination therapy had a higher risk for injection-site-related events, with an RR of 1.85. Therefore, with the extension of follow-up time, in addition to pharmacological tolerance, lower compliance may also lead to a decrease in the efficacy of combination therapy. However, further research is needed to support this hypothesis.

Some studies had demonstrated that gastrointestinal (GI) in nature was the most common adverse events associated with GLP-1RAs; mainly diarrhoea, nausea and vomiting (Jendle et al., 2016; Trujillo and Goldman, 2017). A previous study (Guo et al., 2020) reported that the incidence of GI events was similar between the combination therapy group and the control group. However, another study (Patoulias et al., 2019) demonstrated that there was a significant increase in the risk for nausea and a non-significant increase in the risk for diarrhoea between the combination therapy and SGLT-2i monotherapy. The results of our analysis showed that the risks for diarrhoea and vomiting in GLP-1RA/SGLT-2i combination therapy were all significantly higher than their monotherapy. Therefore, the addition of a GLP-1RA may lead to a higher risk for GI.

As mentioned above, GLP-1RA/SGLT-2i combination therapy may be a better choice for T2DM patients who has poor adequate glycaemic control with monotherapy, and those who wish to lose weight or control SBP. And the effect of weight loss is more obvious for short-term use. However, some adverse events, such as hypoglycaemia, injection-site-related events and GI, are more likely to occur in combination therapy, and they should be alert and closely monitored in clinical practice.

A previous meta-analysis, including 5 RCTs and 6 non-RCTs, investigated the efficacy and safety of GLP-1RA/SGLT-2i combination therapy in T2DM or obesity (Guo et al., 2020), and demonstrated that the risk for cardiovascular events (including myocardial infarction, stroke and heart failure hospitalization) was lower with combination therapy. In addition, compared to the monotherapies, there was no increase in the risk for genital infection in combination therapy. However, in our study, we found that the risk for cardiovascular events (including coronary artery disease, angina pectoris, unstable angina, myocardial infarction, atrial fibrillation, bradycardia, palpitations and tachycardia) with combination therapy was similar to that with monotherapies, and in the subgroup analysis, the risk for genital infection was higher when an SGLT-2i was added to GLP-1RA monotherapy. Two previous meta-analyses (Patoulias et al., 2019; Guo et al., 2020), including 3 and 4 RCTs respectively, investigated the efficacy and safety of GLP-1RA/SGLT-2i combination therapy versus SGLT-2i monotherapy. One of them showed that the levels of TC and LDL-C were decreased in the combination therapy group, while the other revealed that the lipid levels was similar between combination therapy and monotherapy. Nevertheless, our meta-analysis demonstrated that there was no significant difference in TG, TC or HDL-C levels between combination therapy and monotherapy, and the levels of LDL-C were significantly reduced with combination therapy. Another previous study (Mantsiou et al., 2020) performed that no significant differences on the body weight loss was observed in combination therapy compared with SGLT-2i monotherapy when using the long-term data. But based on the results of our analysis, the body weight loss was greater in GLP-1RA/SGLT-2i combination therapy whether compared to GLP-1RA monotherapy or compared to SGLT-2i monotherapy. Retrospective studies have shown that the combination therapy provided better control on HbA1c levels and body weight, nevertheless, the results for blood pressure control were inconsistent. Some studies (Saroka et al., 2015; Kim et al., 2021) demonstrated that both SBP and DBP decreased significantly in combination therapy, whereas some studies (Deol et al., 2017; Díaz-Trastoy et al., 2020) showed that there were no significant differences on them between combination therapy and monotherapy. Another study (Gorgojo-Martínez et al., 2017) indicated that combination therapy can lower SBP better, while there were no significant changes in DBP were found, and these results are consistent with our findings. We believe that the differences in results may be due to the more rigorous inclusion criteria for the present meta-analysis and the more comprehensive inclusion of all relevant and available studies. In addition, this is the major advantage of our study.

There were also some limitations in our meta-analysis. First, the heterogeneity of the results can be considered high, which may be due to the limited number of relevant studies that were included and the small sample size of the incorporated patients. Second, although a subgroup analysis of the added and different classes of experimental drugs was performed, it is not sufficient, the heterogeneity of some efficacy outcomes was still high, so further research is needed. As the drugs of the control groups or the background therapies were not specifically defined in some of the included articles, and only one compared the effects of high and normal doses of experimental drugs, we were unable to perform a more detailed subgroup analysis according to them. Third, some of the results were pooled from only two included articles, such as those for DBP and TC, and more data are needed to support these results. Finally, only one paper examined the effects of these drugs at week 104, and there are limited data on the long-term effects. Thus, more data from long-term follow-up studies are needed.

## 5 Conclusion

In conclusion, compared to monotherapy, GLP-1RA and SGLT-2i combination therapy can more effectively improve blood glucose levels, reduce weight, and control systolic blood pressure in patients with type 2 diabetes. Although GLP-1RA and SGLT-2i combination therapy is associated with a higher risk for some adverse events, such as hypoglycaemia and injection-site-related events, no severe adverse events were found. This meta-analysis provides essential evidence that may guide clinical management decisions for T2DM.

## Data Availability

The original contributions presented in the study are included in the article/Supplementary Material, further inquiries can be directed to the corresponding author.

## Abbreviations

GLP-1RA, glucagon-like peptide-1 receptor agonists; SGLT-2i, sodium-glucose co-transporter-2 inhibitors; T2DM, type 2 diabetes mellitus; RCTs, randomized controlled trials; NIDDM, non-insulin-dependent diabetes mellitus; AACE, American Association of Clinical Endocrinologists; ADA, American Diabetes Association; PRISMA, Preferred Reporting Items for Systematic Reviews and Meta-Analyses; HbA1c, glycated hemoglobin; FPG, fasting plasma glucose; AEs, adverse events; BMI, body mass index; SBP, systolic blood pressure; DBP, diastolic blood pressure; 2h PG, 2h postprandial glucose; SMD, standardized mean difference; CIs, confidence intervals; SD, standard deviation; SE, standard error; LDL-C, low-density-lipoprotein cholesterol; TG, triglycerides; TC, total cholesterol; HDL-C, high-density-lipoprotein cholesterol; GI, gastrointestinal.
