# Supplementary material for: The Efficacy and Safety of the Combination Therapy With GLP-1 Receptor Agonists and SGLT-2 Inhibitors in Type 2 Diabetes Mellitus: A Systematic Review and Meta-analysis
Source: Front Pharmacol. 2022 Feb 4;13:838277. doi: 10.3389/fphar.2022.838277 (PMC8854770; doi:10.3389/fphar.2022.838277)
Supplement: Supplementary file 1 [file DataSheet1.DOCX]

A)


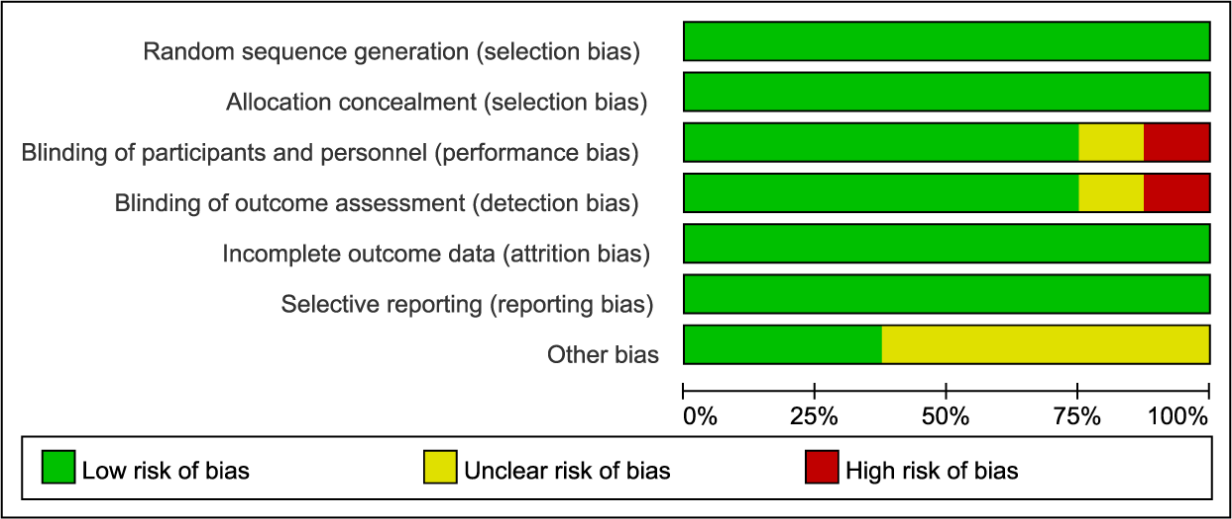


B)


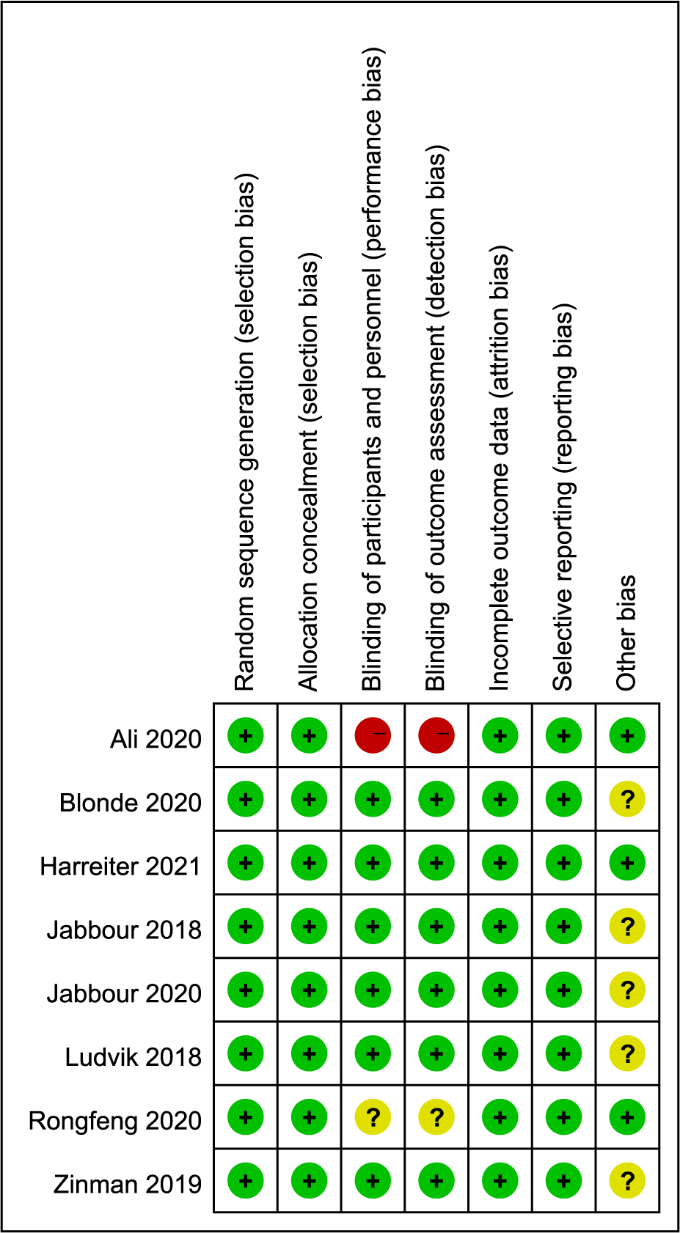


**Fig. 1S** **A)** Risk of bias of the included studies. **B)** Risk of bias summary of the included studies.


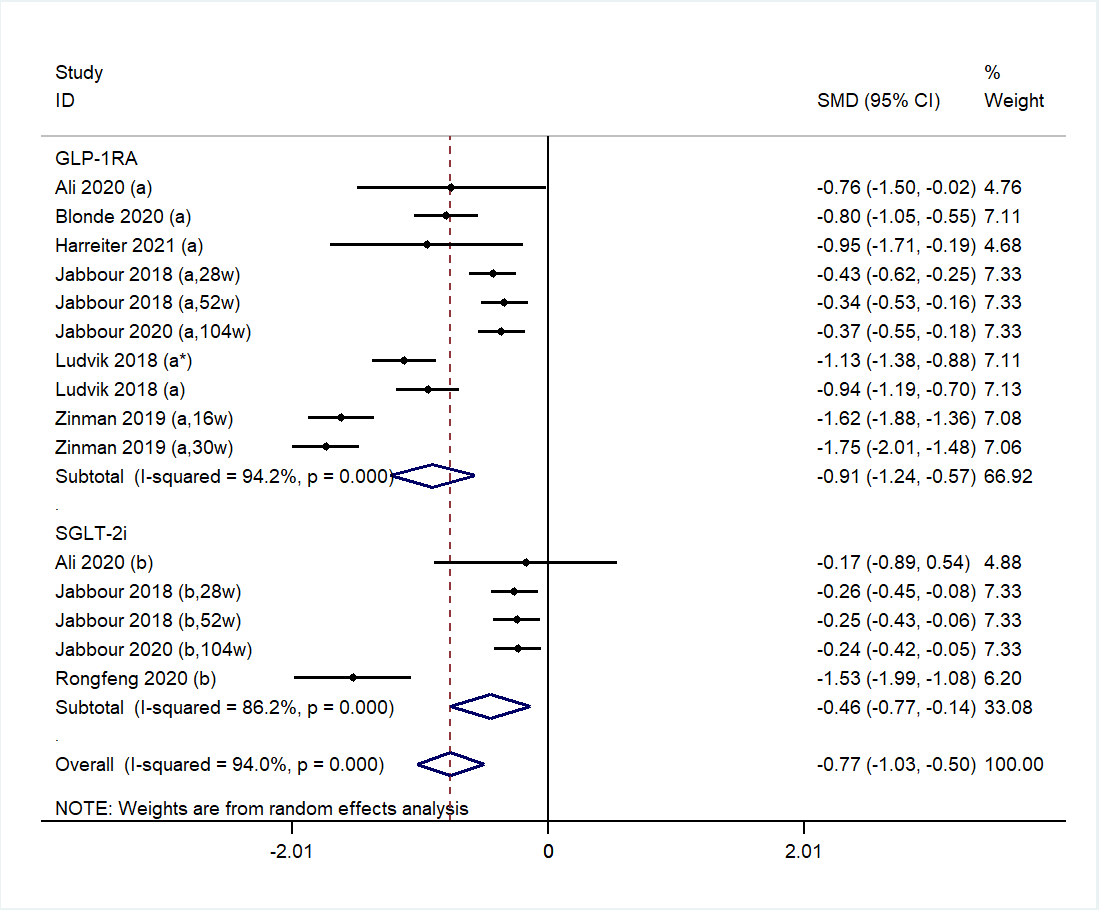


**Fig.2S** Forest plot of the changes in HbA1c between the combination therapy of GLP-1RA and SGLT-2i and their monotherapy. a: add-in drug, GLP-1RA; b: add-in drug, SGLT-2i; *: add-in drug, high-dose.


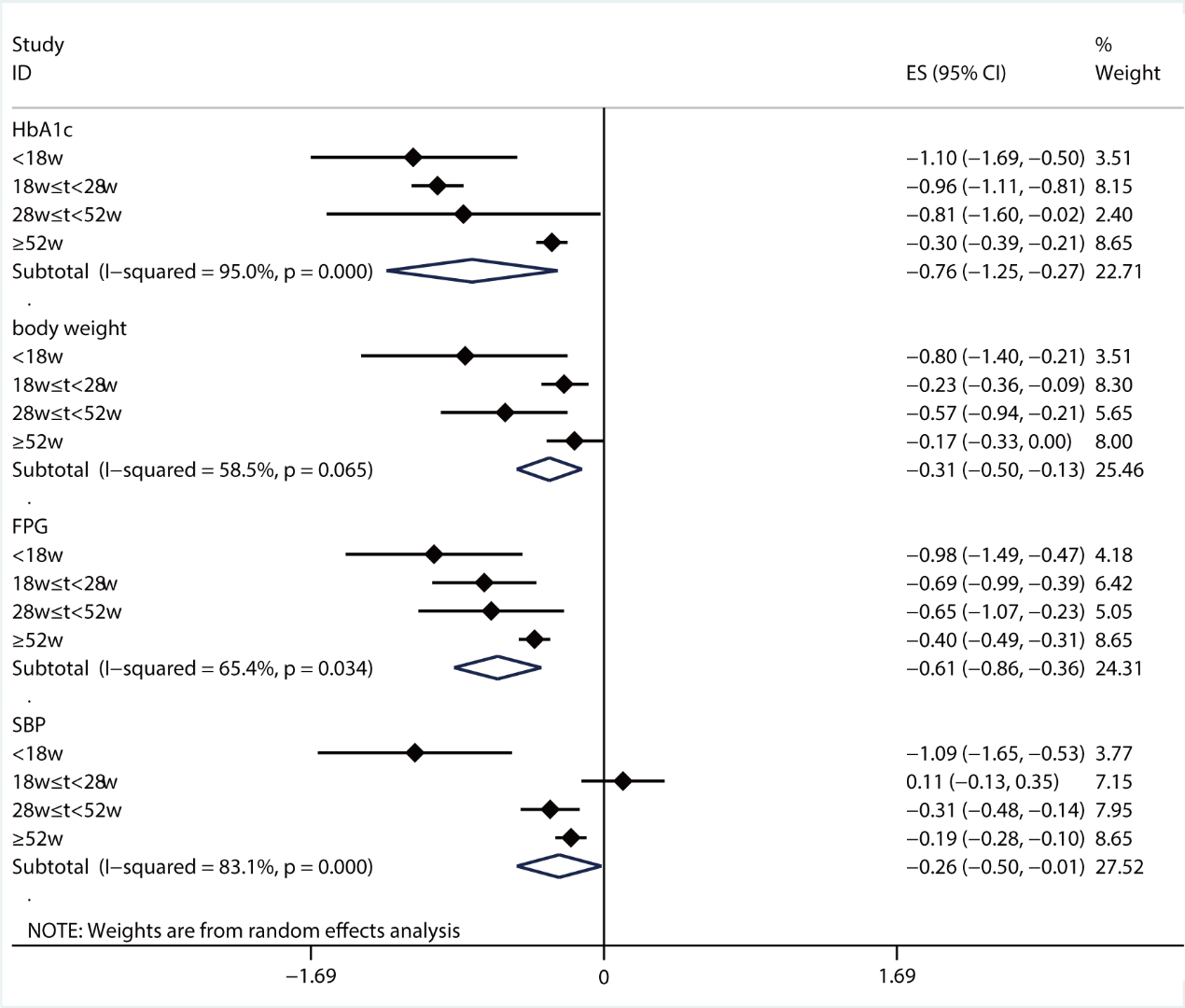


**Fig. 3S** Forest plot of the mean changes in HbA1c (%), body weight (kg), FPG (mmol/L) and SBP (mmHg) between the combination therapy of GLP-1RA and SGLT-2i and their monotherapy by week.


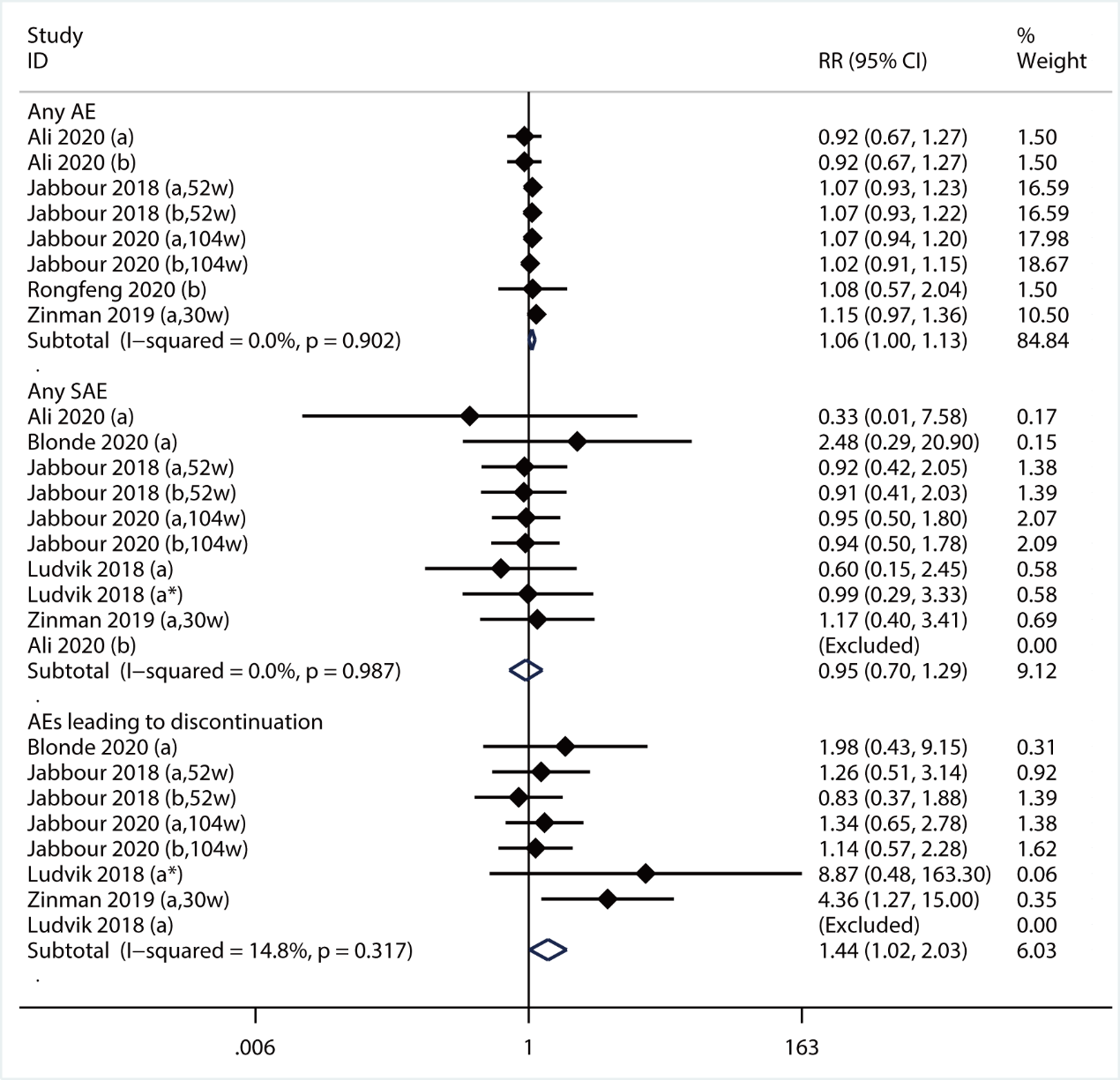


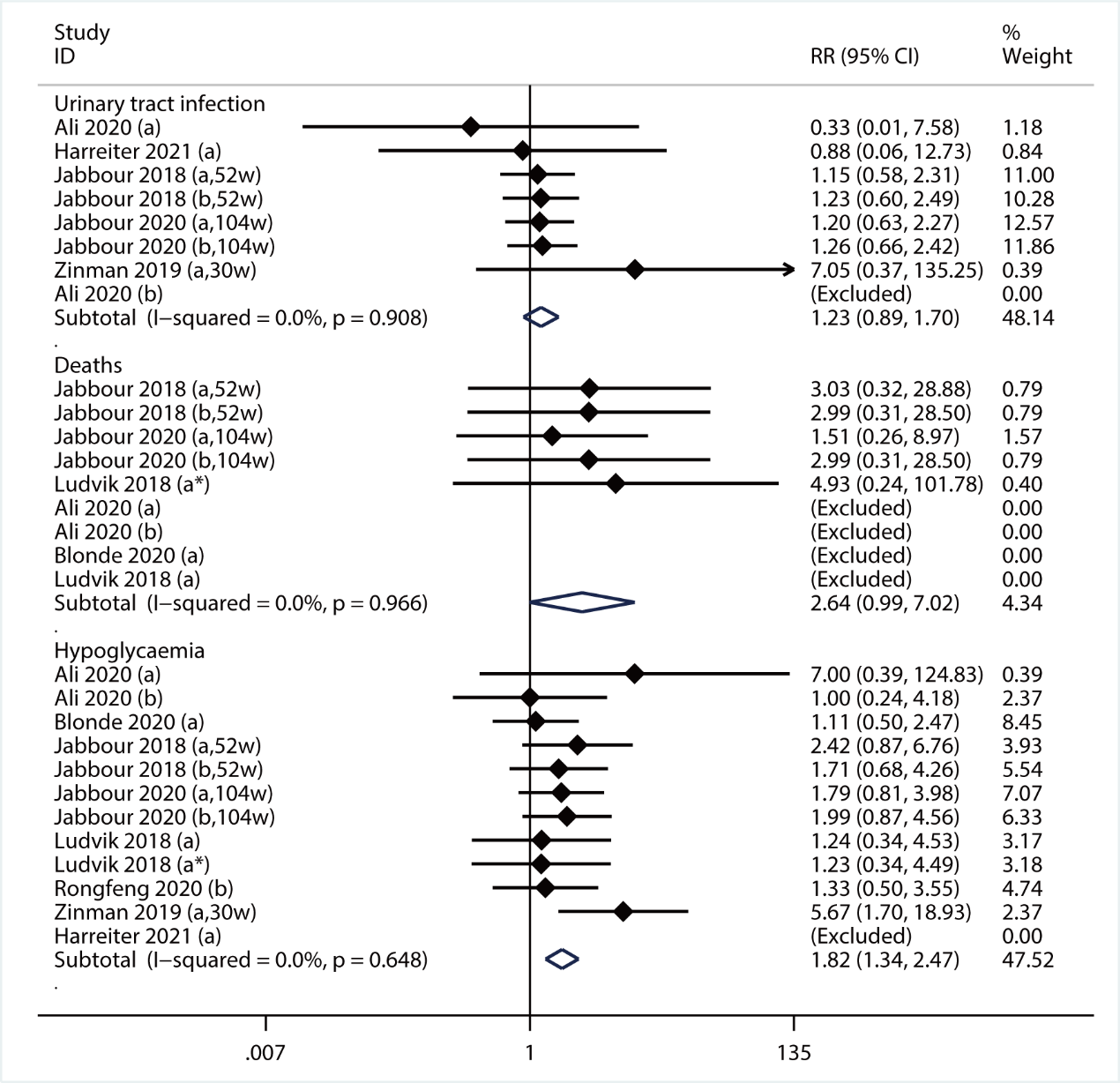


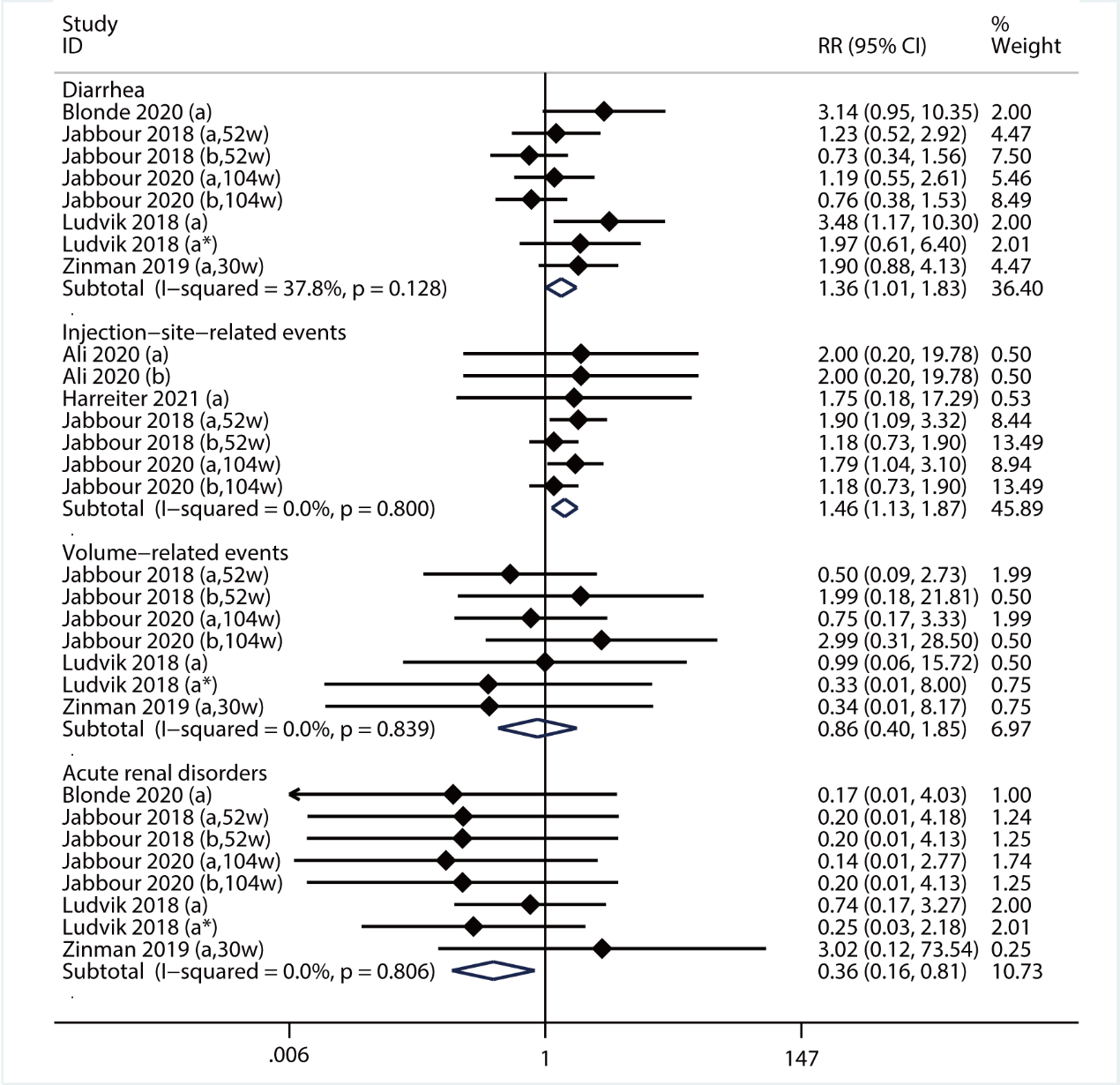


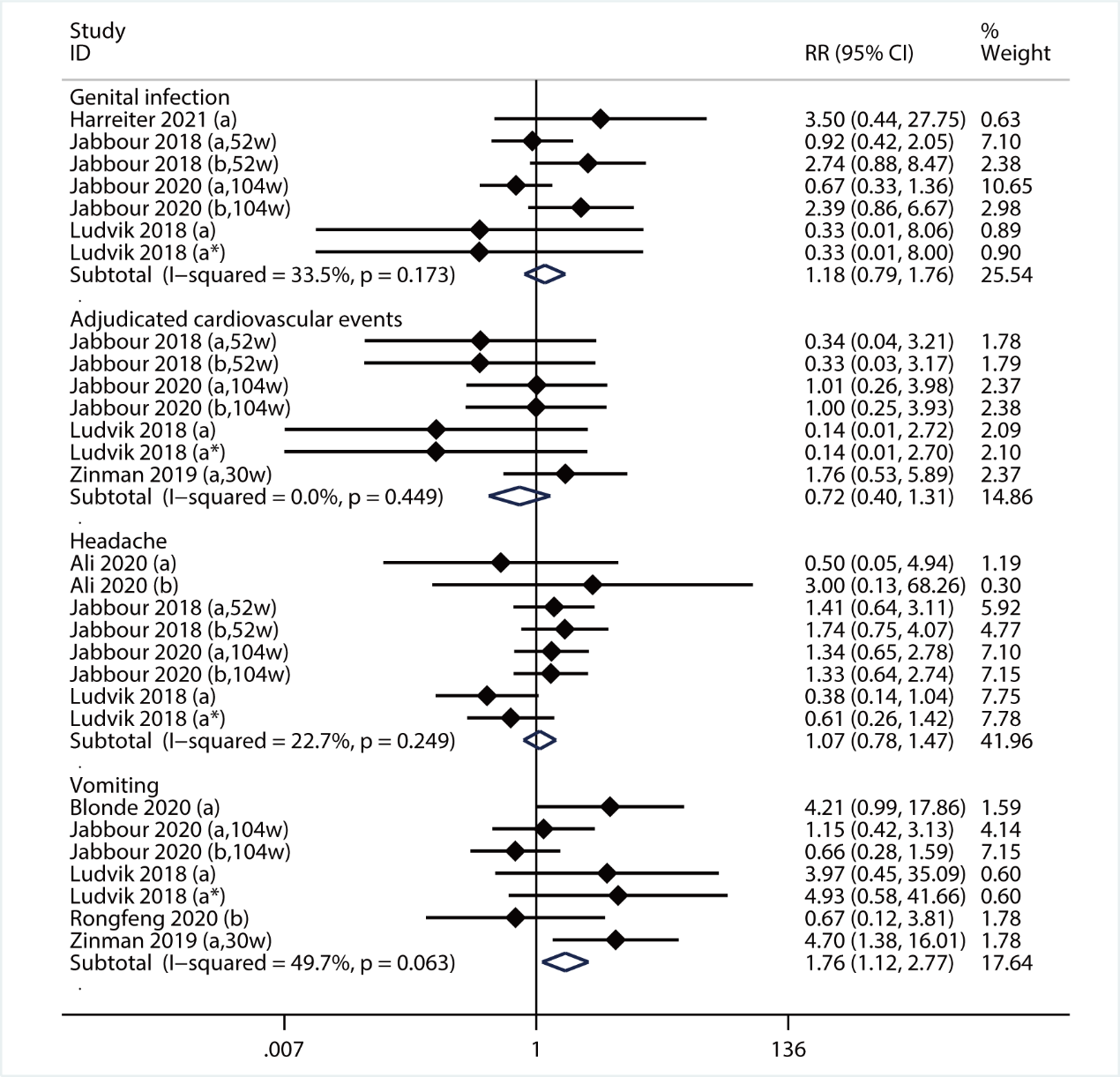


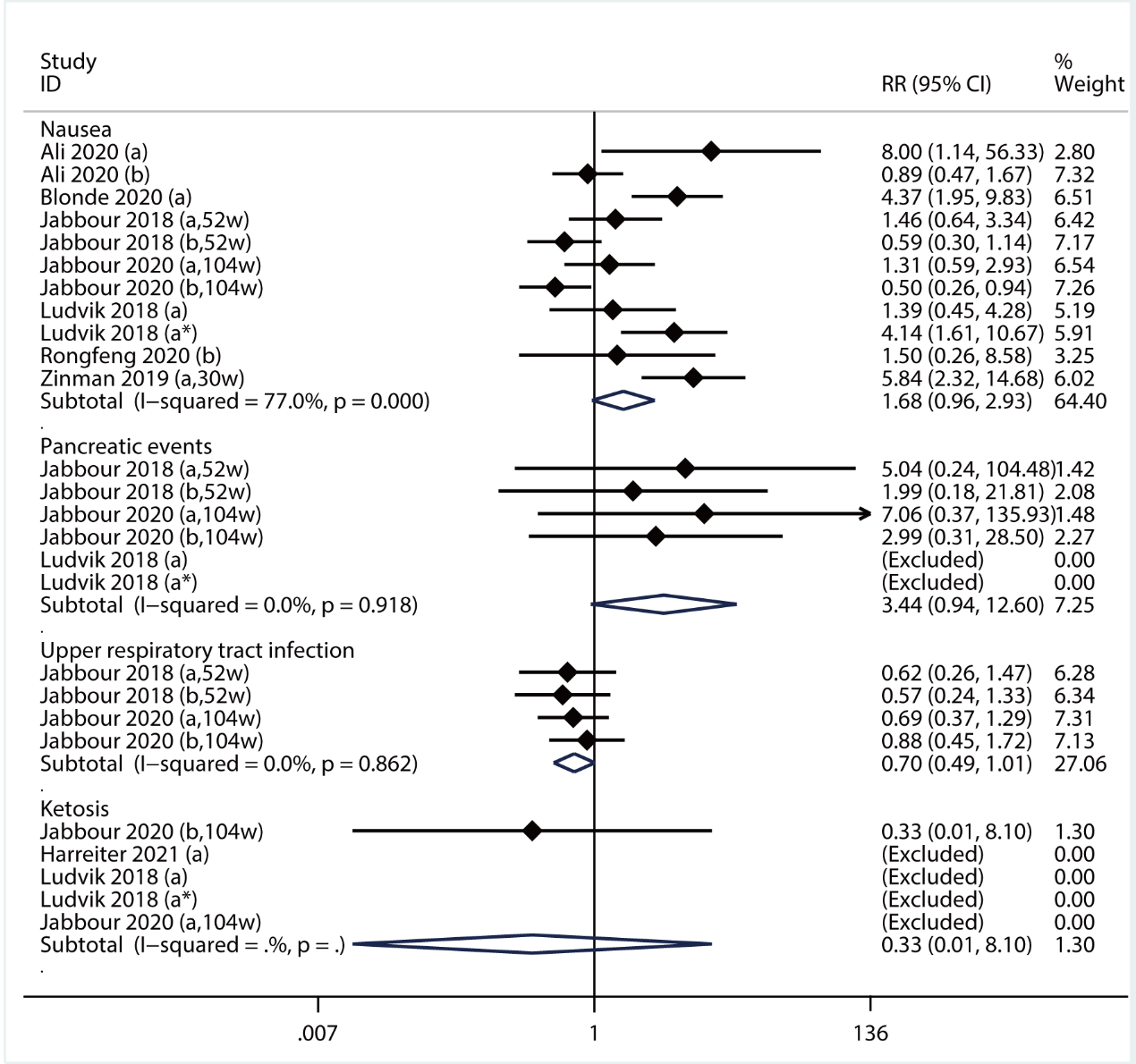


**Fig.4S** Forest plot of all the considered adverse events between the combination therapy of GLP-1RA and SGLT-2i and their monotherapy.

**Table 1S** Total results of Egger’s test for all considered efficacy measures that considered of the included studies.

|  |  |
| --- | --- |
| Outcomes | p |
| HbA1c | 0.115 |
| Body weight | 0.117 |
| FPG | 0.260 |
| SBP | 0.144 |
| 2h PG | 0.087 |
| BMI | 0.664 |
| HDL-C | 0.925 |
| LCL-C | 0.197 |
| TG | 0.805 |
| Waist circumstance | 0.708 |
|  |  |
